# A Combined Pharmacophore Modeling, 3D QSAR and Virtual Screening Studies on Imidazopyridines as B-Raf Inhibitors

**DOI:** 10.3390/ijms160612307

**Published:** 2015-05-29

**Authors:** Huiding Xie, Lijun Chen, Jianqiang Zhang, Xiaoguang Xie, Kaixiong Qiu, Jijun Fu

**Affiliations:** 1 Department of Chemistry, Yunnan University, Kunming 650091, China; E-Mails: front701228.student@sina.com (H.X.); zjq1986526@21cn.com (J.Z.); 2 Department of Chemistry, School of Pharmaceutical Science & Yunnan Key Laboratory of Pharmacology for Natural Products, Kunming Medical University, Kunming 650500, China; E-Mails: chenglijun1414@126.com (L.C.); fujj920@163.com (J.F.)

**Keywords:** imidazopyridine, B-Raf inhibitors, 3D QSAR, pharmacophore, virtual screening

## Abstract

B-Raf kinase is an important target in treatment of cancers. In order to design and find potent B-Raf inhibitors (BRIs), 3D pharmacophore models were created using the Genetic Algorithm with Linear Assignment of Hypermolecular Alignment of Database (GALAHAD). The best pharmacophore model obtained which was used in effective alignment of the data set contains two acceptor atoms, three donor atoms and three hydrophobes. In succession, comparative molecular field analysis (CoMFA) and comparative molecular similarity indices analysis (CoMSIA) were performed on 39 imidazopyridine BRIs to build three dimensional quantitative structure-activity relationship (3D QSAR) models based on both pharmacophore and docking alignments. The CoMSIA model based on the pharmacophore alignment shows the best result (q^2^ = 0.621, r^2^_pred_ = 0.885). This 3D QSAR approach provides significant insights that are useful for designing potent BRIs. In addition, the obtained best pharmacophore model was used for virtual screening against the NCI2000 database. The hit compounds were further filtered with molecular docking, and their biological activities were predicted using the CoMSIA model, and three potential BRIs with new skeletons were obtained.

## 1. Introduction

Cancer as the second cause of mortality is a major health problem all over the world [1], so it is still important to discover new anticancer drugs in spite of the progress in medicine. The Ras-Raf-MEK-ERK pathway, also called ERK/MAP or MAPK kinase pathway, is important for cell proliferation and survival, and its hyper-activation has been reported in up to 30% of human cancers [2,3,4]. Raf kinase exists as three isoformsin this pathway: A-, B-, and C-Raf. B-Raf kinase has been identified as the primary activator [5], and the mutations in B-Raf kinase have been observed in approximately 7% of human cancers, with a different frequency in a variety of human cancers, such as melanoma (50%–70%), ovarian (35%), thyroid (30%), and colorectal (10%) cancers [6]. Therefore, B-Raf kinase has recently emerged as an important and exciting target in cancer treatment [7,8,9].

Pharmacophore modeling can offer a valuable insight into interactions between receptors and ligands. A pharmacophore model reveals the ensemble of steric and electrostatic characteristics of different compounds, by which new classes of inhibitors can be discovered when one class of inhibitors is found. Therefore, pharmacophore searching is a good way to find various chemical structures with the same features in a virtual screening [10,11,12].

Quantitative structure-activity relationship (QSAR) methods have been successfully employed to assist the design of new small molecule drug candidates and explore the ligand-protein interaction mechanism [13,14,15,16,17,18,19,20]. Comparative molecular field analysis (CoMFA) and comparative molecular similarity indices analysis (CoMSIA) are two of the most widely used 3D QSAR methodologies. Lennard-Jones and Coulomb potential functions are introduced by CoMFA to calculate the energies of steric and electrostatic interactions between the compound and the probe atom, and the obtained results can be represented as a three-dimensional “coefficient contour” map [21]. However, in order to avoid some inherent deficiencies caused by Lennard-Jones and Coulomb potential functions, Gaussian function is used by CoMSIA to calculate the energies of interactions between the compound and the probe atom, and the obtained coefficient contour maps can show how steric fields, electrostatic fields, hydrophobic fields, hydrogen bond donor (HBD) and hydrogen bond acceptor (HBA) influence the activity of inhibitors [22].

Recently, a series of imidazopyridines as B-Raf inhibitors (BRIs), shown in Table 1, have been reported in the literature [23]. To understand the structural basis for inhibitory activity and design potent inhibitors, pharmacophore models were created and 3D QSAR studies were performed for these imidazopyridines by using CoMFA and CoMSIA based on both pharmacophore and docking alignments. In addition, in order to discover new classes of BRIs, the obtained pharmacophore model was used as a 3D query for virtual screening against the NCI2000 database. The hit compounds were further filtered by molecular docking, and the biological activities of the hit compounds were predicted by the obtained 3D QSAR model.

**Table 1:** Chemical structures and bioactivity values of the imidazopyridines in the current study.

Compound	General Structure	Substituents	IC_50_ (nM)	pIC_50_
1	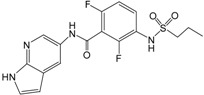	-	42	7.377
2 ^b^	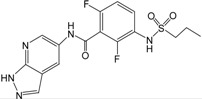	-	18	7.745
3	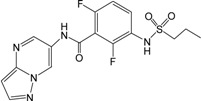	-	247	6.607
4 ^a^	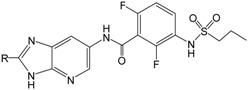	H	61	7.215
5		Me	40	7.398
6		Et	59	7.229
7 ^a^		*i*-Pr	60	7.222
8		*t*-Bu	69	7.161
9 ^b^		Cyclobutyl	31	7.509
10		4-Piperidine	107	6.971
11		3-Piperidine	167	6.777
12	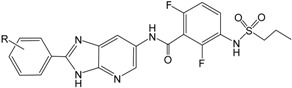	H	3.6	8.444
13		4-F	4.4	8.357
14 ^a^		4-Cl	2.2	8.658
15		4-Br	2.2	8.658
16 ^a^		3-F	3.1	8.509
17		3-Cl	1.1	8.959
18 ^b^		3-Br	0.76	9.119
19		2-F	8.0	8.097
20		2-Cl	27	7.569
21 ^a^		2-Br	27	7.569
22		3,4-di-F	3.4	8.469
23		3,4-di-Cl	1.5	8.824
24 ^b^		4-MeO	1.1	8.959
25 ^a^		4-Me	1.3	8.886
26 ^b^		4-CF_3_	1.4	8.854
27		4-CF_3_O	2.4	8.620
28 ^a^		4-CN	2.7	8.569
29		4-MeSO_2_	1.4	8.854
30		3-MeO	1.2	8.921
31 ^b^		3-CF_3_	1.0	9.000
32 ^a^		4-Pyridyl	3.2	8.495
33		3-Pyridyl	3.0	8.523
34 ^b^	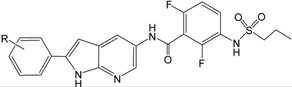	H	1.0	9.000
35 ^a^		4-F	1.1	8.959
36		4-Cl	2.4	8.620
37 ^b^	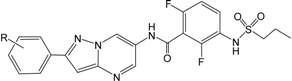	H	4.6	8.337
38		4-F	11	7.959
39 ^a^		4-Cl	8.2	8.086

^a^ Test set compounds; ^b^ Compounds used to generate pharmacophore models.

## 2. Results and Discussion

### 2.1. Pharmacophore Generation

**Figure 1:**
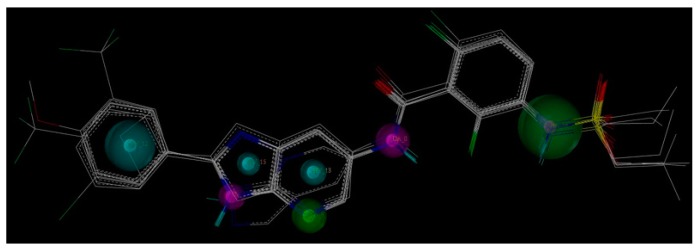
The selected GALAHAD model includes two acceptor atoms (green), three donor atoms (magenta) and three hydrophobes (cyan). The sphere sizes indicate query tolerances.

After the Genetic Algorithm with Linear Assignment of Hypermolecular Alignment of Database (GALAHAD) run, twenty pharmacophore models were generated with default parameters, for which statistical values are listed in Table 2. Among the conflicting demands of maximizing pharmacophore consensus, maximizing steric consensus, and minimizing energy, each obtained model represents a different tradeoff. As shown in Table 2, each model has Pareto rank 0, which implies no one model is superior to any other one. Model_12 has a high energy, which is due to steric clashes [24]. The good models should have small value of energy, high values of Specificity, N_hits, Sterics, H-bond and Mol_Qry [25]. Therefore, Model_06 was considered to be the best model and its statistical values are listed in Table 2. This model was not only used for the molecular alignment to produce CoMFA and CoMSIA models in the 3D QSAR studies, but also was converted into a UNITY query for virtual screening studies. As shown in Figure 1, this model contains two acceptor atoms, three donor atoms and three hydrophobes, and the nitrogen atom attached to –SO_2_– group acts both acceptor and donor atoms.

**Table 2:** The statistical values of pharmacophore models after GALAHAD run.

No.	Specificity	N_hits	Features	Pareto Rank	Energy	Sterics	H-Bond	Mol_Qry
Model_01	4.37	8	10	0	11.15	3574.20	1683.50	561.51
Model_02	2.93	8	11	0	19.35	3488.20	1822.20	381.41
Model_03	1.57	8	13	0	11.63	3390.50	1685.10	703.76
Model_04	1.34	8	12	0	18.07	3287.20	1854.60	319.43
Model_05	0.24	8	8	0	15.77	3370.20	1791.80	282.04
**Model_06**	**4.96**	**8**	**8**	**0**	**28.49**	**3242.80**	**1901.20**	**439.41**
Model_07	3.10	8	10	0	19.61	3365.90	1761.60	479.45
Model_08	−0.15	8	10	0	35.19	3639.30	1767.30	367.28
Model_09	0.11	8	8	0	48.42	3312.90	1775.00	562.73
Model_10	2.11	8	11	0	25.32	3130.60	1806.20	445.97
Model_11	3.53	8	8	0	13.04	3727.10	1786.60	165.73
Model_12	4.37	8	10	0	144.90	3504.50	1757.90	399.20
Model_13	3.40	8	13	0	10.95	2673.80	1743.10	583.52
Model_14	2.48	8	8	0	10.03	2992.50	1768.10	249.76
Model_15	4.30	8	10	0	9.35	3189.60	1715.70	235.40
Model_16	2.06	8	12	0	13.21	2832.40	1762.00	445.41
Model_17	3.12	7	10	0	6.94	3084.10	1587.70	304.97
Model_18	3.34	8	9	0	17.22	3054.40	1746.00	423.70
Model_19	−0.01	8	9	0	13.59	3423.70	1699.30	225.07
Model_20	4.82	8	9	0	6.82	2660.50	1576.80	355.02

The selected model (Model_06) is indicated in boldface.

### 2.2. 3D QSAR Studies

**Figure 2:**
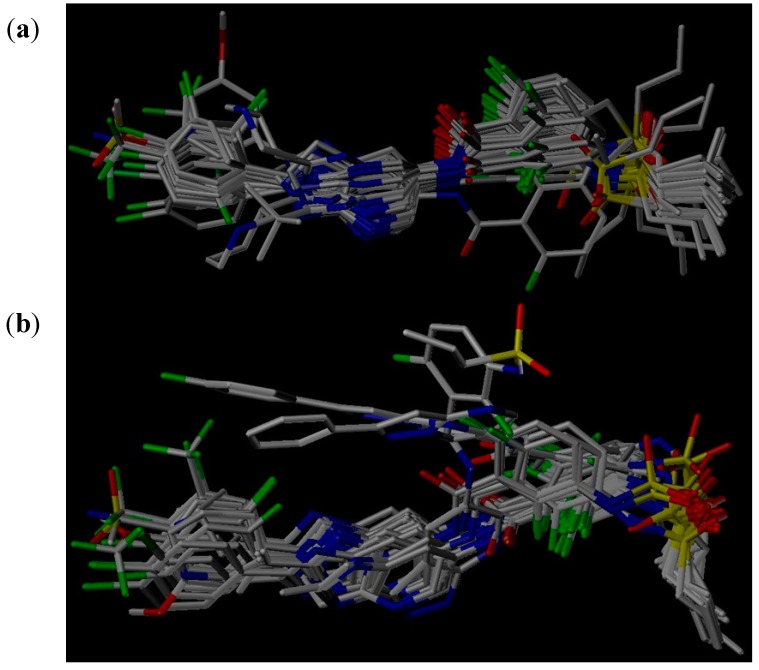
(**a**) Pharmacophore-based alignment of the total data set; and (**b**) Docking-based alignment of the total data set.

The structural alignment of compounds is a crucial step in the development of successful 3D QSAR models. In order to obtain reasonable results, all compounds of the data set were aligned according to both pharmacophore and molecular docking to derive CoMFA and CoMSIA models in current study. Figure 2a,b show pharmacophore-based and docking-based alignments of all the 39 molecules used in 3D QSAR models, respectively.

#### 2.2.1. CoMFA and CoMSIA Statistical Results

In order to get an effective 3D QSAR model, a series of statistical parameters, cross-validated coefficient (q^2^/r^2^_cv_), standard error estimate (SEE) and F-statistic values (F) were calculated as defined in SYBYL. The CoMFA and CoMSIA statistical results based on both pharmacophore-based and docking-based models are shown in Table 3, which shows that the pharmacophore-based modeling yielded q^2^(r^2^_cv_) = 0.501 for CoMFA model and q^2^(r^2^_cv_) = 0.621 for CoMSIA model, while the docking-based modeling gave q^2^(r^2^_cv_) = 0.690, for CoMFA model, q^2^(r^2^_cv_) = 0.541 for CoMSIA model, respectively.

**Table 3:** Summary of CoMFA and CoMSIA statistical results.

Components	Pharmacophore-Based Model		Docking-Based Model	
	CoMFA	CoMSIA	CoMFA	CoMSIA
q^2^(r^2^_cv_)	0.501	0.621	0.690	0.541
SEE	0.185	0.063	0.019	0.312
*F* value	113.846	410.567	3206.612	47.971
r^2^_pred_	0.786	0.885	0.590	0.607
No. of compounds	29	29	29	29
No. of optimal components	4	10	14	3
**Contributions**				
Steric	0.579	0.196	0.542	0.185
Electrostatic	0.421	0.201	0.458	0.185
Hydrophobic	-	0.291		0.338
H-bond donor	-	0.161		0.165
H-bond acceptor	-	0.151		0.127

#### 2.2.2. Validation of 3D QSAR Models

In order to validate the 3D QSAR models, the predictive correlation (r^2^_pred_) was used to assess the predictive abilities of the CoMFA and CoMSIA models from the test set (Table 1) which was not included in the generation of the models. As shown in Table 3, the pharmacophore-based models exhibit better predictive ability than the docking-based models, where the pharmacophore-based modeling yielded r^2^_pred_ = 0.786 for CoMFA model and r^2^_pred_ = 0.885 for CoMSIA model, while the docking-based modeling gave r^2^_pred_ = 0.590 for CoMFA model and r^2^_pred_ = 0.607 for CoMSIA model, respectively.

**Figure 3:**
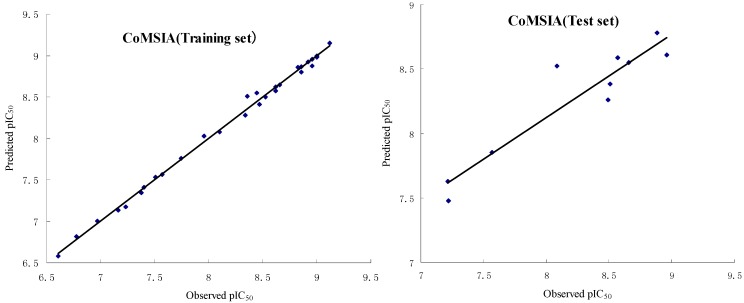
Plots of observed *vs.* predicted activities of the training set and test set molecules from CoMSIA analysis.

We mainly focus on the CoMSIA obtained from pharmacophore-based alignment due to its satisfactory statistical results and its best predictive ability. As shown in Table 3, this CoMSIA model has a q^2^(r^2^_cv_) of 0.621 with ten optimal components, SEE of 0.063 and F value of 410.567, which indicates it is a quite good model. The corresponding field contributions of steric, electrostatic, hydrophobic, HBD and HBA are 0.196, 0.201, 0.291, 0.161 and 0.151, respectively, which suggests that each field gives similar contribution to activity. The observed and predicted pIC_50_ by the CoMSIA model of the training and test sets are given in Table 4, and the correlations between the observed and predicted pIC_50_ of training and test sets are depicted in Figure 3.

**Table 4:** Observed and predicted pIC_50_ of the training and test sets from the CoMSIA model.

Compound	Observed pIC_50_	Pharmacophore-Based CoMSIA	
		Predicted pIC_50_	Residual
1	7.377	7.343	0.034
2	7.745	7.761	−0.016
3	6.607	6.583	0.024
4 ^a^	7.215	7.630	−0.415
5	7.398	7.410	−0.012
6	7.229	7.173	0.056
7 ^a^	7.222	7.478	−0.256
8	7.161	7.137	0.024
9	7.509	7.534	−0.025
10	6.971	7.004	−0.033
11	6.777	6.817	−0.040
12	8.444	8.549	−0.105
13	8.357	8.511	−0.154
14 ^a^	8.658	8.551	0.107
15	8.658	8.645	0.013
16 ^a^	8.509	8.384	0.125
17	8.959	8.956	0.003
18	9.119	9.149	−0.030
19	8.097	8.076	0.021
20	7.569	7.563	0.006
21 ^a^	7.569	7.850	−0.281
22	8.469	8.412	0.057
23	8.824	8.860	−0.036
24	8.959	8.872	0.087
25 ^a^	8.886	8.782	0.104
26	8.854	8.802	0.052
27	8.620	8.572	0.048
28 ^a^	8.569	8.588	−0.019
29	8.854	8.863	−0.009
30	8.921	8.923	−0.002
31	9.000	8.996	0.004
32 ^a^	8.495	8.259	0.236
33	8.523	8.500	0.023
34	9.000	8.976	0.024
35 ^a^	8.959	8.608	0.351
36	8.620	8.618	0.002
37	8.337	8.283	0.054
38	7.959	8.030	−0.071
39 ^a^	8.086	8.521	−0.435

^a^ Test set compounds.

#### 2.2.3. CoMSIA Contour Maps

**Figure 4:**
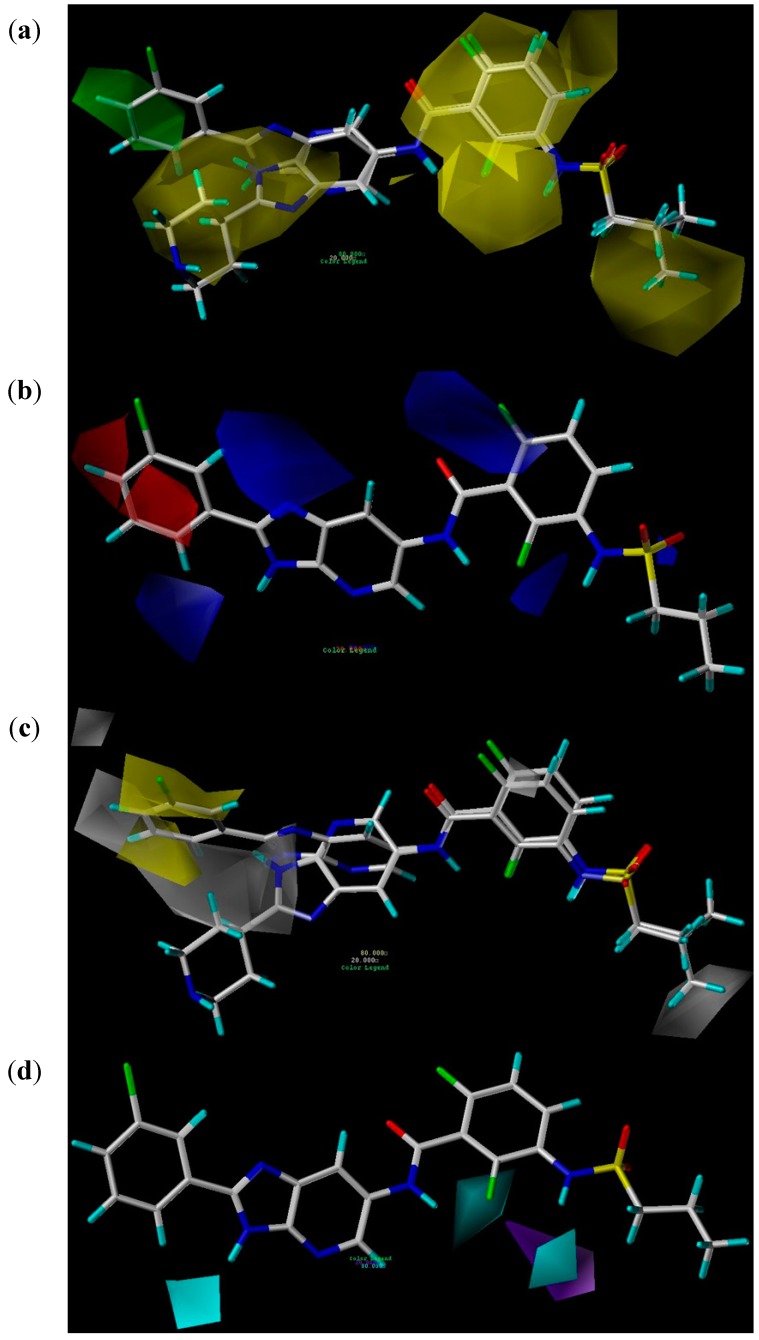

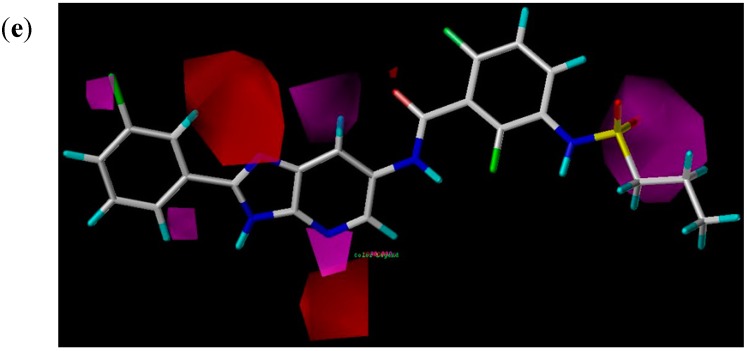
(**a**) Steric contour maps in combination with compounds **18** and **10**: green contours refer to sterically favored regions; yellow contours indicate sterically disfavored areas; (**b**) Electrostatic contour maps in combination with compound **18**: blue contours refer to regions where positively charged substituents are favored; red contours indicate regions where negatively charged substituents are favored; (**c**) Hydrophobic contour maps in combination with compounds **18** and **10**: yellow contours indicate regions where hydrophobic substituents are favored; white contours refer to regions where hydrophilic substituents are favored; (**d**) HBD contour map in combination with compound **18**: cyan contours indicate HBD substituents in this region are favorable to activity; purple contours represent that HBD groups in this area are unfavorable; and (**e**) HBA contour maps in combination with compound **18**: magenta contours show regions where HBA substituents are expected; red contours refer to areas where HBA substituents are unexpected.

CoMSIA not only calculates steric and electrostatic fields as in CoMFA, but also additionally computes hydrophobic, HBD and HBA fields. The CoMSIA contour maps of steric, electrostatic, hydrophobic, HBD, and HBA fields are revealed in Figure 4a–e. Compound 18 and compound 10 were selected to be superimposed into the contour maps because compound 18 is the most active compound in all 39 imidazopyridines and compound 10 is the least active compound in 30 compounds (compounds 4–33) in which there is a substituent group attached to the imidazole ring. For each field, the favorable and disfavored contours represent 80% and 20% level contributions, respectively.

The steric contour map with compounds 18 and 10 is shown in Figure 4a, in which green contours refer to sterically favored regions, while yellow contours indicate sterically disfavored areas. A large green contour near the phenyl group attached to the imidazole ring of compound 18 indicates that a bulky group in this region is favorable to bioactivity. It is confirmed by the fact that compounds 12–39 with bulky substitution in this region have higher bioactivity than compounds 1–11 with no substitution. A large yellow contour near the piperidine group attached to the imidazole ring of compound 10 suggests that a bulky group in this area is unfavorable to bioactivity. This is supported by the lower activity of compounds 10–11 with large substituents in this area, compared with the higher activity of compounds 4–9 with small substituents.

The electrostatic contour map with compound 18 is shown in Figure 4b, where a negative potential is favorable to activity in the red areas while a positive potential is favorable in the blue areas. A red contour near the 4-position of the benzene ring and a blue contour near the 2-position of the benzene ring suggest that an electronegative atom on the 4-position of the benzene ring can increase the bioactivity while an electronegative atom on the 2-position of benzene ring is able to reduce the bioactivity. This can be validated by the higher activity of compounds 13, 14 and 15, compared with the lower activity of compounds 19, 20 and 21, respectively. Figure 4b also shows that a blue contour is near the nitrogen atom of the imidazole ring, which means that the nitrogen atom is unfavorable to activity. This is supported by the fact that compounds 12 and 13 with a nitrogen atom at that position are less active than compounds 34 and 35 with no nitrogen atom, respectively.

The hydrophobic contour map in combination with compounds 18 and 10 is shown in Figure 4c, in which yellow contours refer to regions where hydrophobic groups are favored while white contours represent areas where hydrophilic groups are favored. Two large yellow contours near the phenyl group attached to the imidazole ring of compound 18 means that a hydrophobic group in this region is favorable to bioactivity while a large white contour near the piperidine group attached to the imidazole ring of compound 10 suggests that a hydrophobic group in this area is unfavorable. This can be seen by the fact that compounds 12–39 with a benzene ring near the yellow contour have higher bioactivity while compounds 5–11 with a hydrophobic group in white contour have lower bioactivity. Figure 4c also displays that there is a yellow contour near the 3-position of benzene ring, which hints that a hydrophilic group on the 3-position of benzene ring is able to decrease the bioactivity. This can be proved by the descending activities of compounds 18, 17 and 16.

The HBD contour map in combination with compound 18 is shown in Figure 4d, where cyan contours indicate that HBD groups in this area are favorable to activity while purple contours represent that HBD substituents in this region are unfavorable. Three cyan contours are close to the three –NH groups of the compound 18, which suggests the necessity of the hydrogen atoms at these positions for high bioactivity. This can be validated by the fact that compounds 12–36 with three –NH groups have higher bioactivity than the other compounds with two –NH groups.

The HBA contour map in combination with compound 18 is shown in Figure 4e, in which magenta and red contours represent areas where HBA substituents are favored and disfavored, respectively. Two large magenta contours are located near the oxygen atoms of –CO– and –SO_2_– groups and one large magenta contour is next to the nitrogen atom of the pyridine ring, which implies that the two oxygen atoms and one nitrogen atom are HBA atoms. Figure 4e also shows there is a large red contour near the nitrogen atom of the imidazole ring, which indicates that the nitrogen is unfavorable to the activity. This is proved by the lower activity of compounds 12 and 13 with a nitrogen atom, compared with the higher activity of compounds 34 and 35 with no nitrogen atom, respectively, which is consistent with the result derived from electrostatic contour map.

### 2.3. Virtual Screening

#### 2.3.1. Pharmacophore Model Validation

**Figure 5:**
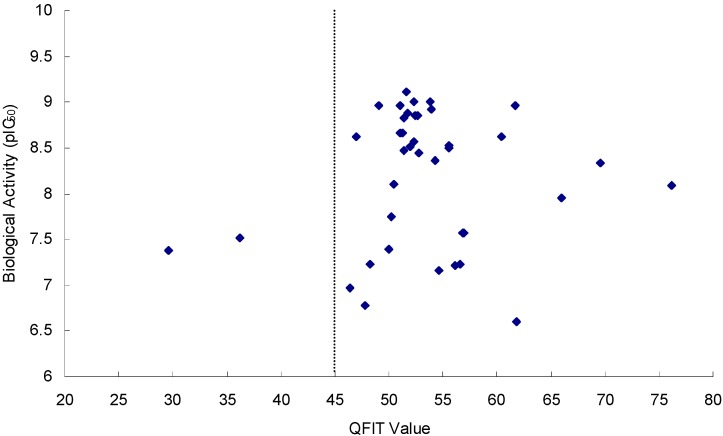
Plots of QFIT values *vs.* biological activity (pIC_50_ values) of 39 inhibitors.

In order to validate the pharmacophore model in the virtual screening, the QFIT (pharmacophoric match between query and the hit compound) values of all the 39 imidazopyridine inhibitors were tested by using the obtained best pharmacophore model (Model_06), and the correlations between the QFIT values and pIC_50_ values of the 39 inhibitors are depicted in Figure 5. It can be seen that 37 of 39 inhibitors have high QFIT values (QFIT > 45), which indicates that the Model_06 is a potent pharmacophore model in the virtual screening. In order to screen the dataset effectively, the QFIT values of hit compounds were set to more than 45 in this study.

#### 2.3.2. Docking Model Validation

**Figure 6:**
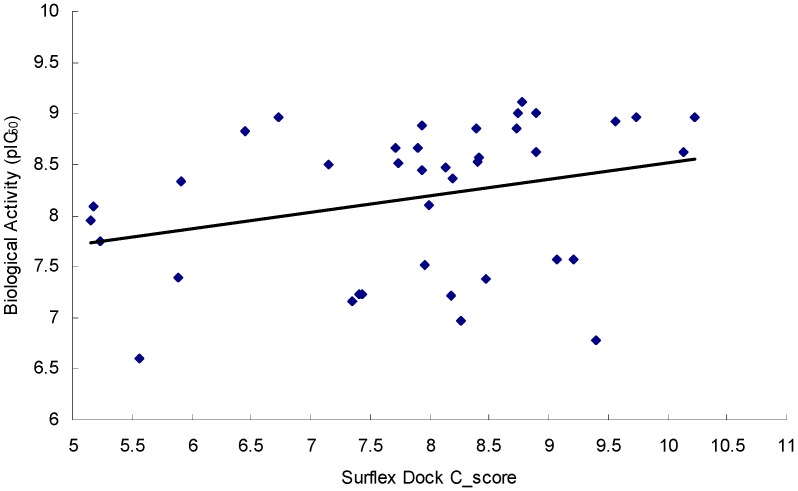
Plots of C_score values *vs.* biological activity (pIC_50_ values) of 39 inhibitors.

In order to validate the docking model in the virtual screening, the C_score values of all the 39 imidazopyridine inhibitors were assessed by using Surflex-Dock, and the correlations between the C_score values and pIC_50_ values of the 39 inhibitors are depicted in Figure 6. It can be shown that all the 39 inhibitors have high C_score values (C_score > 5.0), which suggests that the protomol generated by Surflex-Dock is an effective docking model in the virtual screening. In order to screen the dataset effectively, the C_score values of hit compounds were set to more than 5.0 in this study.

#### 2.3.3. Screening of NCI2000 Database

The obtained best GALAHAD model (Model_06) was converted into an UNITY query, which was screened against NCI2000 database (234,054 compounds). The “3D Search” option was implemented to perform virtual screening, primary filters such as Lipinski’s rule of five and Van der Waals bumps were applied to reduce the dataset [26], and the QFIT values of hit compounds were set to more than 45. The screening of the pharmacophore query yielded eight hit compounds that met the specific requirements. The eight hit compounds were further subjected to molecular docking by using the Sulflex-Dock. Three compounds were selected based on the docking C_score values (C_score > 5.0). The pIC_50_ values of the three hit compounds were predicted by the obtained CoMSIA model based on pharmacophore alignment. Chemical structures and predicted pIC_50_ values of the three hit compounds are listed in Table 5. The three hit compounds show quite good predicted pIC_50_ values (pIC_50_ > 7.6), which are expected to design novel BRIs with new skeleton.

**Table 5:** Chemical structures and predicted activity values of the hit compounds.

Hit Compound	Structure	QFIT Value	Docking C_Score	Predicted pIC_50_
NCI 94680	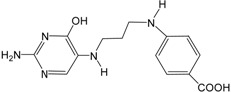	66.50	6.84	8.520
NCI 527880	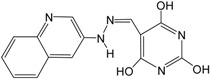	67.58	5.55	8.263
NCI 183519	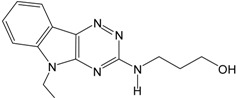	62.80	5.28	7.667

## 3. Experimental Section

### 3.1. Compounds and Biological Data

A series of imidazopyridines as BRIs were used for this study, and their structures and bioactivity values are shown in Table 1 [23]. The pIC_50_ (−log IC_50_) values were used to derive 3D QSAR models. The whole data set of 39 compounds was divided into two groups in an approximate ratio of 3:1; a training set with 29 compounds, and a test set with 10 compounds (Table 1). The selection of the training and test sets was performed manually such that high, moderate and low activity compounds were selected in roughly equal proportions in both sets. The training set was used to build predictive models, while the test set was used to validate the predictive ability of the models.

### 3.2. Molecular Modeling

The SYBYL 7.3 molecular modeling package from Tripos Inc., St. Louis, MO, USA, which was installed on Red Hat Linux workstations [27], was used to perform 3D QSAR modeling analyses, calculations and visualizations. Identification of the bioactive conformation is a very crucial step in a 3D QSAR study [28], so the conformation of compound **4** was obtained from the protein data bank (PDB code: 4MBJ) [23], where the compound **4** is combined with B-Raf kinase. The compound **4** was modified by adding hydrogen atoms after extracted from the complex, and then minimized with the following three steps: (i) using Steepest Descent with initial optimization of 200 simplex iterations in the condition of Tripos force field and Gasteiger-Marsili charge to optimize the energy; (ii) using conjugate gradient to optimize the energy; and (iii) using BFGS to optimize the energy [29]. Each conformation of other inhibitors was constructed based on the compound **4**, and energy minimizations were performed according to the above procedure.

### 3.3. Pharmacophore Hypothesis

The pharmacophore hypothesis includes two main stages: firstly, the ligands are aligned to each other in internal coordinate space; secondly, the produced conformations are aligned in Cartesian space, which was performed by using GALAHAD module of SYBYL. The features used to generate the pharmacophore model include HBD atoms, HBA atoms, hydrophobic and charged centers [30,31,32]. Eight compounds with high activity (Table 1) were selected to create the pharmacophore hypothesis in the current study, and the conformers for all molecules were generated by genetic algorithm.

### 3.4. Molecular Docking

The molecular docking was carried out by using the Surflex-Dock module of SYBYL, and all parameters were set with default values in the whole process. All the molecules were docked to the binding site of B-Raf kinase crystal structure in complex with compound **4** (PDB code: 4MBJ) [23]. Before docking, the ligand was extracted, all the water molecules were removed and hydrogen atoms were added to the receptor. The protomol was generated using the docking based method; with the ligand location in the same coordinate space in the receptor. In our study, each conformer of all 39 inhibitors was docked into the binding site 10 times and the C_score values were used to evaluate the docking analysis. The top ranked conformations for each molecule were extracted and aligned together for the subsequent 3D QSAR study [33]. The Surflex-Dock was also used to filter the hit compounds in the virtual screening.

### 3.5. Molecular Alignment

The 3D molecular alignment plays a very important role in 3D QSAR studies, and affects the outcome of the CoMFA and CoMSIA statistical analysis. There are three main different molecular alignments for 3D QSAR: maximum common substructures overlap, pharmacophore-based alignment and docking-based alignment [29]. In order to get reasonable results, both of the pharmacophore-based and docking-based alignment procedures were performed in our study. Pharmacophore-based alignment was performed using GALAHAD and docking-based alignment was done by using Surlflex-Dock.

### 3.6. CoMFA and CoMSIA Models

CoMFA models use a Lennard-Jones potential to calculate steric fields and a Coulombic potential to compute electrostatic fields. During the calculation of CoMFA fields, a 3D cubic lattice with grid spacing of 2.0 Å in three-dimensional directions was generated by SYBYL, and the grid pattern stretched 4.0 Å in all directions of each molecule. A sp^3^ carbon probe atom as steric probe and a +1.0 charge as an electrostatic probe were taken to calculate the probe-ligand interaction energies at each lattice point. The electrostatic contributions were ignored at lattice points with maximal steric interaction and the cut-off for energies was set to ±30 kcal/mol [21]. However, five different similarity fields (steric, electrostatic, hydrophobic, HBD, and HBA) were computed in CoMSIA models. The same lattice box as in CoMFA was used to derive CoMSIA models, in which a probe of charge +1, a radius of 1, hydrophobicity and hydrogen bonding properties of +1 were use to calculate the five fields, and an attenuation factor was set to 0.3 for the Gaussian distance-dependent function [22].

### 3.7. Statistical Analysis

The pIC_50_ values were used as dependent variables and CoMFA and CoMSIA descriptors as independent variables in the 3D QSAR models. The optimal number of components was obtained according to q^2^(r^2^_cv_) when the partial least squares (PLS) method with cross-validation (leave-one-out) was used in SYBYL. Based on the obtained optimal number of components, the final model was generated with the training set after a PLS analysis was performed with no validation and column filtering 2.0. The quality of the 3D QSAR models can be evaluated by the obtained q^2^ and the predictive capability of the models can be determined by r^2^_pred_. The predicted activities for the test set were obtained from the model produced by the training set.

## 4. Conclusions

B-Raf kinase has proven to be an important target for treatment of cancers. In order to design and search for potent BRIs, a combined pharmacophore modeling, 3D QSAR and virtual screening studies on imidazopyridines were performed. Pharmacophore models were derived from eight compounds with high activity and diverse structure by using GALAHAD, and the best pharmacophore model obtained included two acceptor atoms, three donor atoms and three hydrophobes. 3D QSAR techniques based on both pharmacophore and docking alignments, CoMFA and CoMSIA, were applied for the 39 imidazopyridine BRIs. The CoMSIA model obtained from the pharmacophore-based alignment showed the best result (q^2^ = 0.621, r^2^_pred_ = 0.885), and the CoMSIA contour maps indicated that the phenyl group attached to the imidazole ring and –NH, –CO–, –SO_2_– groups in imidazopyridine molecules can increase the inhibitory activity, while the nitrogen atom of the imidazole ring may reduce the inhibitory activity. In addition, the best pharmacophore model obtained was used for virtual screening against the NCI2000 database, and eight hit compounds were obtained. Three compounds were selected using molecular docking, which showed perfect predicted pIC_50_ values. The present pharmacophore modeling, 3D QSAR, and virtual screening approach provides useful information to design and synthesize novel BRIs.
